# Science Priorities for Seamounts: Research Links to Conservation and Management

**DOI:** 10.1371/journal.pone.0029232

**Published:** 2012-01-18

**Authors:** Malcolm R. Clark, Thomas A. Schlacher, Ashley A. Rowden, Karen I. Stocks, Mireille Consalvey

**Affiliations:** 1 National Institute of Water & Atmospheric Research, Wellington, New Zealand; 2 University of the Sunshine Coast, Maroochydore, Australia; 3 University of California San Diego, La Jolla, California, United States of America; University of Aberdeen, United Kingdom

## Abstract

Seamounts shape the topography of all ocean basins and can be hotspots of biological activity in the deep sea. The Census of Marine Life on Seamounts (CenSeam) was a field program that examined seamounts as part of the global Census of Marine Life (CoML) initiative from 2005 to 2010. CenSeam progressed seamount science by collating historical data, collecting new data, undertaking regional and global analyses of seamount biodiversity, mapping species and habitat distributions, challenging established paradigms of seamount ecology, developing new hypotheses, and documenting the impacts of human activities on seamounts. However, because of the large number of seamounts globally, much about the structure, function and connectivity of seamount ecosystems remains unexplored and unknown. Continual, and potentially increasing, threats to seamount resources from fishing and seabed mining are creating a pressing demand for research to inform conservation and management strategies. To meet this need, intensive science effort in the following areas will be needed: 1) Improved physical and biological data; of particular importance is information on seamount location, physical characteristics (e.g. habitat heterogeneity and complexity), more complete and intensive biodiversity inventories, and increased understanding of seamount connectivity and faunal dispersal; 2) New human impact data; these shall encompass better studies on the effects of human activities on seamount ecosystems, as well as monitoring long-term changes in seamount assemblages following impacts (e.g. recovery); 3) Global data repositories; there is a pressing need for more comprehensive fisheries catch and effort data, especially on the high seas, and compilation or maintenance of geological and biodiversity databases that underpin regional and global analyses; 4) Application of support tools in a data-poor environment; conservation and management will have to increasingly rely on predictive modelling techniques, critical evaluation of environmental surrogates as faunal “proxies”, and ecological risk assessment.

## Introduction

Seamounts are prominent components of the seascapes of all ocean basins [1]. These raised topographical features and the ecosystems which they support, have historically been viewed as unique, diverse and productive systems embedded in a more homogeneous deep-sea environment [e.g.], [ 2]–[4].

Research on seamounts has recently been focused in a field programme as part of the Census of Marine Life (CoML)–The Census of Marine Life on Seamounts (“CenSeam”) [5]–[6]. CenSeam brought together scientists working in the fields of seamount ecology, taxonomy, conservation, fisheries, geology, physical oceanography, and informatics. Census funding catalyzed two main areas of activity: 1) enhanced collaboration amongst the scientific communities of numerous countries encompassing multiple disciplines, and 2) an expansion of studies to regional and global scales that enabled research of greater generality and scope to address key ecological hypotheses.

The expansion of research effort beyond national programmes, coupled with the ability to plan and carry out research at broader geographic scales, substantially advanced our understanding of how seamounts are structured, how they function as ecosystems, and how human activities impact on them. This progress is evident across a range of fields. For example, descriptions of many new species added to the stock of knowledge on seamount biodiversity, as did numerous scientific papers published on seamount oceanography, ecology, and the vulnerability and management of seamount resources [4], [7]–[8].

Five major scientific summaries of seamount ecology have been published in the last 5 years: 1) a book bringing together contributions on seamount geology, ecology, and fisheries [9], 2) a critical evaluation of commonly held views on seamount ecological structures, processes and drivers [10], 3) a detailed review of the state of knowledge of seamounts in terms of oceanographic processes and settings, biological mechanisms (e.g. trophic transfers), connectivity, and impacts from fishing [1], 4) a compilation of synoptic papers focussed on the geology and geophysics of seamounts [11], and 5) scientific output from the CenSeam programme itself contained in a special issue of Marine Ecology [8]. The Marine Ecology issue papers address key aspects of seamount ecology and human impacts, and it contains a thorough critique of existing ‘paradigms’ about seamounts [4]. Since then, further studies associated with CenSeam have been completed and a number have been published in a thematic collection of PLoS ONE (Marine Life on Seamounts – The CenSeam Collection (2012) PLoS Collections: http://www.ploscollections.org/CenSeam). Taken together, these papers form the basis to assess where seamount research has been in the last few decades, where it is currently, and where it should head to address conservation and management needs in the future.

In this paper we review recent seamount research, evaluate how well some key ecological aspects of seamounts are understood, and highlight where gaps remain that need to be filled to improve the robustness and uptake of scientific advice for environmental management of seamounts. We restrict our list to research that, in our view and from our experience, is both achievable and of the highest priority for conservation and sustainable use of deep-sea seamount ecosystems.

## Analysis

### 1. Key seamount results

To evaluate where future research priorities should lie, it is useful to briefly review some of the main findings of CenSeam and other seamount research in recent years that are relevant to the management of seamounts.

#### 1.1 Seamounts are generally not isolated habitats with a highly endemic fauna

Because most seamounts are geographically isolated topographic features, separated from other seamounts by deep water and considerable distance, it seems logical to equate them with oceanic islands (sensu MacArthur & Wilson [12] and Hubbs [13]). However, a growing number of studies suggest seamounts are generally not ecologically isolated or island-like systems, and they can, and often do, have assemblages of similar species composition to those found in adjacent deep-sea habitats on the continental slope or banks e.g. [14]–[17]. Nevertheless, despite such similarity in species composition, seamount assemblages can have a different structure in terms of the abundance or frequency of species [18]–[20].

Connectivity between seamounts is a key element affecting the degree of isolation or similarity of seamount populations. Recent studies indicate connectivity is highly variable, but there are reports of considerable genetic linkages among populations of invertebrates on distant seamounts [21]–[24]. Traditionally, high levels of endemism had been reported from seamounts e.g. [3], [25], [26], but it is unclear how sampling effort may have biased these estimates. Conversely, more recent studies indicate that rates of endemism may not be elevated on seamounts [16], [27]–[30].

#### 1.2 Seamounts are heterogeneous habitats

Seamounts span a broad depth range, are influenced by diverse oceanographic processes, are situated in diverse geological settings, and comprise heterogeneous habitat types. Thus, the concept of seamounts as a single, relatively well-defined habitat type appears outdated, giving way to a growing recognition that within-seamount variability can be high, and seamounts differ substantially across a range of spatial scales. For example, environmental parameters that vary with depth are a major driver of species composition on seamounts [31] as elsewhere in the deep sea [32]. Similarly, seafloor type and character (e.g. substratum, hardness, composition, mobility), and the complexity of habitat arrays are key determinants of species occurrence, distribution and diversity in the benthos of both shallow and deep marine habitats [33]–[35]. Such faunal-habitat associations clearly operate on seamounts [16], [36], which is well illustrated by the small-scale distribution of corals which cluster on hard substratum on raised topographical features where currents are strongest [37]. Volcanic activity, lava flows and areas of hydrothermal venting add to habitat diversity on seamounts [38], [39], creating unique environmental conditions which support specialized species and assemblages [40].

#### 1.3 Communities on seamounts are variable over large spatial scales

The amount of species turnover within seamount fauna varies with spatial scale, with both similarities and differences recorded among sites separated from kilometres [41], [42] to ocean basins [43]. Set against recent biogeographic classifications [44] there is an expectation of marked differences in the biological community composition in different parts of the world. Physical characteristics, water column stratification, and oceanic flow conditions interact on a seamount to provide a number of local dynamic responses that can regulate the spatial scale of faunal distributions. These can include Taylor Columns or Cones, doming of density surfaces, enclosed circulation cells and enhanced vertical mixing [45]. However, variability in background oceanic flow means these processes can also be variable, and there are many ways in which a seamount can alter local oceanographic conditions, and how seamount biological communities can be affected by oceanic currents e.g. [37], [46]–[48].

The distribution of faunal communities on seamounts is affected by different environmental factors and levels of variability [1]. Deep-sea fish assemblages have been shown to be similar between seamounts and the adjacent slope (scales of km) [18], as well as across oceans (1000 s of km) [43]. In the latter case it appears that global-scale circulation of deep-sea water masses is a key component of fish distribution. At the regional scale, similarities in faunal composition between seamounts and other habitats have been reported for galatheids [17] and molluscs [30] in the South Pacific: in both cases, seamounts share a common regional pool of species with the communities of non-seamount habitats. On seamounts along the Vitoria-Trinidade seamount chain off Brazil, the general invertebrate assemblages differ from those on the shelf, yet there is no gradient in species richness with distance offshore along the linear east-west chain [41]. These recent studies emphasise that it is impossible to generalise about the spatial scales over which faunal assemblages of seamounts are structured.

#### 1.4 Seamounts are increasingly exploited by humans

Fishing on seamounts is a widespread activity with a long tradition of exploitation. Seamounts continue to be fished globally, with targeted bottom trawling for deep-sea, commercial species such as orange roughy (*Hoplostethus atlanticus*), pelagic armourhead (*Pseudopentaceros wheeleri*) and alfonsino (*Beryx* spp.) [49]–[51] and for pelagic species such as tunas [52]–[54]. Clark & Tittensor [55] developed an index of risk for seamounts to fishing. This index combined several global and local sources of data to determine the vulnerability of seamounts based on the coincidence of seamount summit location and depth, target fishery ranges, and predicted habitat suitability of seamounts for coral. It then evaluated the potential risk of future exploitation based on the known distribution of fisheries, and hence where seamounts had not already been impacted. The spatial maps showed the most “at-risk” seamounts are spread throughout the world's oceans, with many in areas of the high seas, especially in the South Atlantic, southern Indian, South Pacific, and North Atlantic Oceans [55].

Mining in the deep sea is an emerging environmental issue potentially affecting seamounts and other habitats [56]–[58]. Seamounts have become the focus of exploration for seabed minerals, particularly poly-metallic sulphides in the Southwest Pacific [59] and cobalt-rich crusts in the central Pacific Ocean [60]–[62]. In recent years exploration licences have been granted in offshore waters of several countries in the Southwest Pacific for poly-metallic sulphides, and mining is likely to occur at the Solwara I site off Papua New Guinea in one or two years (http://www.nautilusminerals.com). Additional licence areas in the Indian Ocean have been applied for by China and Russia in 2011. Although seamounts that could host these sulphide deposits are worldwide, an indication of the relative amount of habitat can be gained from the distribution of hydrothermal vents along the back-arc basins of the Southwest Pacific. The known number of vent sites is about 40 (http://www.noc.soton.ac.uk/chess/database/db_home.php) out of over 2000 large seamounts in the region with summit depths less than 2000 m (based on Allain, et al. [63]). Exploration activities for cobalt-rich ferromanganese crust seamounts are not as advanced, although there are about 1200 large seamounts and guyots which fall inside a ‘cobalt crust rich zone’ of commercial potential in the central Pacific region [62].

#### 1.5 Seamounts are affected by human exploitation

Fishery resources on seamounts are susceptible to adverse impacts: the life history characteristics of many seamount fishes make them unproductive e.g. [64], [65], few seamount fisheries have been sustainable [66], and bycatch species also decline rapidly following fishing on seamounts [67], [68].

There have been few specific research studies on the impacts of fishing in the deep sea (see review by Gage, et al. [69]). However, studies on seamounts off Australia and New Zealand have clearly demonstrated significant differences in the structural complexity of benthic habitats, species numbers and abundance, and the composition and structure of assemblages between fished and unfished seamounts [70]–[72]. Large sessile taxa (e.g. sponges, echinoids, cold-water corals) are particularly susceptible to damage, showing dramatic reductions in coverage after only a few trawls [73], [74].

The effects of mining are more uncertain because few studies have been carried out. Mining for poly-metallic sulphides could occur on inactive seamounts, or those with active (but not extreme temperature) hydrothermal vents. Hence impacts at inactive sites could be on “normal” benthic fauna, such as corals and sponges, in which case some of the effects may be similar to bottom trawling (see references above). Creation of sediment plumes in the water column, and discharge of processed material can be additional impacts (see section 4.5). Direct physical impacts at active sites on the spatially restricted hydrothermal vent fauna are likely to be significant initially, with the removal of habitat and a largely endemic fauna [75].

#### 1.6 Seamounts are very slow to recover from impacts

Recovery of vulnerable species, and the assemblages which they form, from human impacts is predicted to be very slow in the deep sea [76]. The expectation of protracted returns (if any) to pre-impact conditions is mainly based on the exceptionally slow growth rates of large, deep-sea megafauna [77]–[79], and variable recruitment due to intermittent dispersal between seamount populations [80]. Williams, et al. [81] examined changes in benthic invertebrate composition on seamounts off Australia and New Zealand following their closure to bottom trawling, and found no signs of recovery after 5–10 years.

In contrast, following disturbance to active hydrothermal areas, regrowth of mineral chimneys may be rapid [82]. A number of studies suggest that re-colonisation, and hence recovery of the dominant vent populations, will probably occur within 5 years [83], [84]. However, this assumes that nearby vent sites remain active and act as sources of recruits to the mined areas [75].

### 2. What science is required for the conservation and management of seamounts?

The exploitation of new marine resources, especially fisheries, has often started without management measures in place. The high seas in particular illustrate how fisheries can develop unchecked, which typically results in overexploitation and stock declines e.g. [66], [85]. Research normally lags behind exploitation, and management further behind again. Hence resource managers are often playing “catch up” and faced with data-poor situations where decisions need to be made without robust or adequate information.

The management and conservation of seamount resources, and seamount habitat, varies in different countries and organisations [76], [86]. However, recent initiatives by the Food and Agriculture Organisation (FAO) for fisheries, the International Seabed Authority (ISA) for mining, and parties to the Convention on Biological Diversity (CBD) and the United Nations General Assembly for more generic conservation, stress a growing need for a more common international approach and information and tools from the science community.

Management approaches for seamounts comprise two complementary categories of management tools: site-specific, and activity-related [76]. Fisheries management is often focussed on the latter, and there is an extensive literature on information needs to conduct stock assessments and manage deep-sea seamount fisheries [51], [87], [88], but ecosystem-based spatial management is currently the main approach called for by international bodies [89].

Some of the key questions that managers may ask in relation to spatial planning include:

1. Where are seamounts located, and what are their physical characteristics?
2. What seamount species may be rare or unique, or particularly vulnerable to the effects of human activities?
3. How connected are seamount ecosystems to enable re-colonisation?
4. How different are seamount communities from one another and from adjacent environments (e.g., continental slope)?
5. What other impacts are there apart from direct physical disturbance?
6. How long may it take for impacted communities to recover?
7. Will climate change and ocean acidification affect faunal communities on seamounts?

Answers to these questions (as well as many others) are usually provided as direct advice in response to individual initiatives that focus on particular geographic areas or on particular human threats. For example, programmes on Vulnerable Marine Ecosystems (VMEs) or Ecologically and Biologically Significant Areas (EBSAs) [89], [90] require specific information on species composition, richness, and vulnerability to human impact (predominantly in the fisheries context). Science can also feed data into risk management frameworks that provide for a more quantitative assessment of risk to various ecological components; this is a tool that managers can use to prioritise the nature and extent of management action [91], [92].

**Figure 1:**
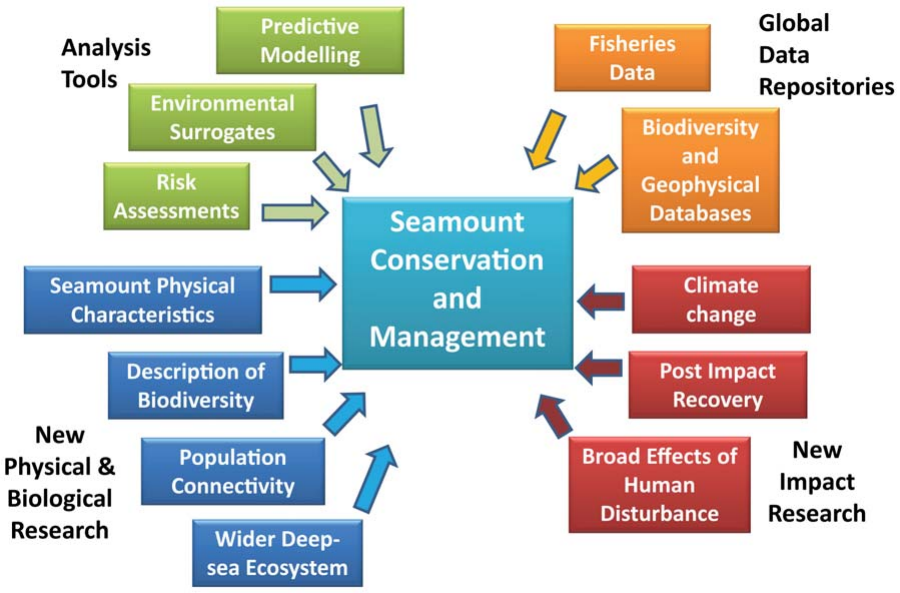
The key areas of research required for improved management and conservation of seamounts over the next decade.

Against the recent findings from seamount research (section 2) and the science needs of environmental managers (this section), the next two sections outline what we regard as priority areas of research for conservation and management over the next decade (section 4), and some of the key data and tools required (section 5). They are either tractable issues using existing data, or are areas where new data and resources are likely to measurably advance seamount conservation and management in a short (5–10 year) time frame (Figure 1).

### 3. Future research needs for seamount conservation and management

In the following sections we summarise some of the key research requirements for future seamount management (and see Table 1).

**Table 1:** Summary of research priorities for seamounts over the next decade based largely on science input required for the growing demands of conservation and management strategies to be developed for seamount ecosystems; no ranking of priorities is implied.

Rationale	Actions	Output(s)
**Seamount locations and physical characteristics**		
Accurate information on location and physical characteristics of seamounts underpins spatial planning approaches	Complement satellite-based predictions of raised topography by direct, ship-based surveys of seafloor.	Seamount locations and attributes better documented over larger geographic areas.
**Description of biodiversity**		
The biodiversity of seamount biota is unknown for most seamounts, and remains incompletely documented in many cases.	Sample in unexplored regions; investigate sampling effort and estimates of species numbers, expand biodiversity inventories; include genetics	More accurate and geographically comprehensive estimates of biodiversity as inputs to conservation planning and management.
**Spatial scales of population connectivity**		
Determining scales of population connectivity among seamounts allows testing key ecological paradigms	Studies on reproductive and larval biology, modelling of particle transport, and genetic structure of populations with depth and distance.	Scales of connectivity among populations of seamount species better known and useful for planning conservation measures.
**Seamounts as part of the deep-sea ecosystem**		
The role of seamounts in supporting species of conservation significance needs comparison with other deep-sea systems.	Expansion of seamount sampling to abutting habitats and ecosystem types using, wherever possible, standardised collection and analysis methods.	Levels of similarity between seamounts and other deep-sea habitats are determined, and indicate the potential for seamounts to act as ‘source’ or ‘sink’ populations
**Broader effects of human disturbance**		
Trawling and mining create sediment plumes. Neither the magnitude nor spatial extent are known.	Determine the nature and magnitude of ecological effects caused by sediment plumes and measure their dispersal and persistence.	Resource managers incorporate such disturbance into mitigation strategies.
Overexploitation of fauna occupying one trophic level is hypothesized to have ecosystem-wide consequences for seamounts	Assess the impact of fishing on large predators, including any implications for food webs and community dynamics.	More ecologically comprehensive assessment of human impacts on seamount ecosystems.
**Recovery dynamics**		
Key rates and metrics of recovery remain unknown for seamount ecosystems.	Determine recovery dynamics of species resilient to physical disturbance, recruitment dynamics, species composition (‘succession’), growth rates of species, genetic connectivity of populations	Environmental managers can establish thresholds of acceptable impact and set time durations for seamount closures to allow recovery.
**Climate change**		
Changes in temperature, chemical composition, circulation patterns and productivity of the world's oceans are occurring. Seamounts may offer sites of “refuge” from such changes.	Determine the ability of different taxa to disperse vertically. Link with studies examining the drivers of species composition/abundance to improve predictions.	Refinement of models to predict changes in faunal distribution with respect to parameters that vary with climate change and ocean acidification (e.g. aragonite saturation horizon).

#### 3.1 Improved seamount location and physical characteristics

A fundamental input to all conservation and management measures is accurate information about the geographic distribution of habitats and their biological resources: all strategies require this spatial knowledge. Hence, there is an urgent need for better bathymetry and identification of topographic features.

Although many areas within EEZs, or those near major shipping lanes, have well-known bathymetry, much of the ocean's seafloor has not been accurately mapped using ship-based techniques, and the topography of most of the global seafloor is predicted from satellite altimetry. Several analyses of seafloor topography, using a range of algorithms, have been carried out to locate seamounts and other similar features such as peaked ridges [93]–[96]. These estimates vary considerably, although the most recent suggest there are about 30,000 large seamounts and over 100,000 smaller knolls [96]. However, the underlying gravity data have limitations, and the algorithms are not always effective and accurate in identifying raised topography; this can result in multiple counts of single seamounts and inaccurate estimates of the summit depth of seamounts. For example, Yesson, et al. [96] compared seamount locations predicted from satellite altimetry with a well-known region around New Zealand. They achieved a 90% match of large (>1 km elevation) seamounts, and a good correlation between predicted and actual summit depth. Conversely, smaller features (250–1000 m elevation) had poor spatial congruence between model and actual locations, and the algorithms tended to define broad ridge peaks as seamounts.

Recent improvements in satellite resolution and algorithms to locate seamounts need to be complemented by direct, ship-board depth measurements. Wessel, et al. [97] suggest that considerable progress could be made by asking ships (especially oceanographic research vessels undertaking trans-ocean passages) to slightly alter course to cover new, or potentially interesting, regions of the seafloor. The Seamount Discovery Tool [98], which allows ship operators to determine whether a proposed ship track will cover a charted or uncharted region, may facilitate better mapping efforts.

#### 3.2 Better descriptions of biodiversity

A good knowledge of biodiversity is required to evaluate whether a seamount has rare or endemic species which may need protection, or a fauna with similarities or differences from other seamounts or habitats that need to be considered in any form of spatial management.

Overall knowledge of species and community composition on seamounts has increased considerably in recent years. However, there are still a number of aspects that require improvement to provide appropriate and adequate information on seamount biodiversity to managers. There is a need to plan sampling to fill gaps from geographic areas or seamount types, especially deep seamounts, and those at high latitudes and in equatorial regions. There are large areas of the South Atlantic, central Pacific, and southern Indian Oceans were few data are available [1]. There have been recent surveys to the mid-Atlantic Ridge off Brazil [99] and to the Southwest Indian Ocean [100], but large gaps remain. The summits of seamounts have also been more intensively sampled than flanks or bases [1]. This is understandable as the summits and upper flanks of seamounts are often where faunal densities are highest for fish, cold-water corals and sponges e.g. [74], [101] yet it gives an incomplete picture of seamount biodiversity.

A second key element of improving knowledge of diversity is to increase the level of sampling during research surveys. In almost all studies of seamount diversity there is a clear pattern that more sampling results in a greater number of species being recorded, whether from individual seamounts [19], [102] or from broader regions [3], [43]. Hence researchers cannot be sure how well the species richness of seamounts is currently described, as sampling effort is uneven. It is very likely that most seamounts are undersampled, and hence species numbers per seamount are underestimated.

More intense sampling is likely to be required in many cases to describe and enable the protection of rare species, or those with a very localised distribution (“spot endemism”) which is an objective of many management agencies. To our knowledge there has been no published evaluation of how estimates of endemism may be affected by sampling effort on seamounts or elsewhere in the sea. However, unpublished modelling by scientists within CenSeam suggests that undersampling will likely result in inflated estimates of rare species (authors' unpublished data). These predictions need to be tested with field data to determine adequate levels of sampling coverage and quantify biases arising from variable sampling effort in seamount surveys.

A third element of future biodiversity description relates to expanding the taxonomic coverage of collections and analyses. To date, the focus of most biological seamount research has been on the larger (e.g. macro- and megafauna) epifauna. Fishes, corals, and crustaceans are the most commonly reported taxa [1], whereas other macrofaunal groups and meiofauna are poorly sampled or identified. Macro-infauna, meiofauna, and bacterial biodiversity may be high on seamounts [103]–[106], and the same aspects that are important, and obvious, for conservation of large megafauna (e.g. diversity, endemism, local population size) apply also to these other elements of faunal communities. Hence a wider range of taxa should be sampled and, most importantly, identified and reported. For some taxa, international efforts have recently resulted in collaboration between taxonomists to ensure that regional or global datasets are accurate (e.g., galatheids [107], stony corals [79]). These sorts of efforts to standardise taxonomic identification and compile regional or global datasets are very important for future analyses of biodiversity patterns, and should be encouraged wherever possible for as many faunal groups and size components as possible.

A further key element of improving biodiversity description is faster identification of species. The recent achievements of the field programmes of the Census of Marine Life in fostering international survey work has highlighted that taxonomists often cannot keep pace with the collection of new species [6]. Variations in identification between taxonomists in different parts of the world, and the uncertain status of operational taxonomic units can make it difficult to undertake robust analyses. Genetic techniques can be used to supplement morphological taxonomy, to ensure accurate specification (separating cryptic species) and potentially rapid processing of samples. A combined approach is to be encouraged [108].

Genetic diversity should be included where possible in evaluating biodiversity. Although genetic techniques are commonly used in taxonomic or connectivity studies, they also give important information on the degree of population differentiation between seamounts [1], [80].

#### 3.3 Spatial scales of population connectivity

The spatial scale of management and conservation depends to a large extent upon the distributional range of species and faunal groups. Spatial patterns of biodiversity, and aspects of population dynamics such as recolonisation and recovery, are strongly driven by biological connectivity between seamounts at a range of temporal and spatial scales. Knowing the level of exchange between seamounts (as well as other habitats) informs managers about the “downstream” effects of impacting one seamount, and the consequences of interrupting this exchange. Recovery from impacts caused by human activities (fishing, mining) depends upon the ability of animals to recolonise an affected area and such re-colonisation will in turn depend on dispersal of organisms from unaffected source populations.

There are numerous factors that can influence dispersal and connectivity between seamount populations. After Clark, et al. [1] these include 1) the physical structure of the oceans (e.g., hydrographic retention mechanisms, currents), 2) environmental conditions affecting development time and larval survival (e.g., temperature, availability of food, presence of predators), 3) spatial separation and the presence of suitable habitat for early life history stages as well as adults, and 4) reproductive mode of fauna, especially for sessile species which disperse only via eggs and larvae compared with mobile species which can, theoreticaly, move between seamounts as juveniles and adults [109]. Variability in oceanographic conditions around seamounts together with differences in biological characteristics means there can be large variations in dispersal distances between species [22], [80].

Clark, et al. [1] and Shank [80] summarised much of the seamount genetic connectivity literature. Most studies have been undertaken on commercially fished species, and have generally shown genetic homogeneity at oceanic or regional geographical scales. The spatial genetic structure of invertebrate species has shown more variable patterns, and appears highly dependent upon reproductive mode. However, many of the connectivity studies have an unbalanced design of sample collection that can make it difficult to separate the effects of distance, depth, and habitat on population connectivity [23], requiring careful survey designs to determine the influence of these factors on dispersal of seamount species [22], [24].

Future studies on connectivity need to involve a combination of morphological and genetic studies, but also consider:

1. Reproductive biology of adults. This needs to include determination of fecundity (as an indicator of how quickly populations could increase given favourable environmental conditions) and in particular spawning characteristics (e.g., brooders or broadcast spawners, planktotroph (feeding) or lecithotroph (non-feeding) larvae) and fertilisation success as these have clearly been shown to be important in the distribution of species [30], [110].
2. Early life history stage ecology. Larval biology, behaviour and ecology are poorly known for most seamount species, fish as well as invertebrates. Their mobility, and distribution by depth, can affect whether they are capable of widespread dispersal, or are likely to remain close to their site of hatching.
3. Modelling of current flows and prediction of patterns of particle advection and transport. The potentially complex nature of current flows on and around seamounts (see summary accounts by White, et al. [45], Clark, et al. [1]) means that it can be difficult to make assumptions about likely dispersal of eggs and larvae. Detailed oceanographic studies over broad spatial scales and density of sampling required may be prohibitively expensive in offshore situations. Modelling of currents (through the water column to allow for vertical migration of species)and the likely spread and direction of particle advection [48], [111], [112] can contribute to understanding the physical dimension of dispersal and improve advice on the spatial extent of management measures.
4. Monitoring of seamount sites for temporal recruitment series. Time series of observations are also needed for verification of the frequency and levels of colonisation events. This could be achieved through, for example, regular monitoring surveys of seamounts using high definition camera equipment able to resolve small-sized recruits on the seafloor, placement and monitoring of settlement plates, and use of in-situ plankton pumps.

#### 3.4 Seamounts evaluated as part of a wider deep-sea ecosystem

Seamounts are one of many habitat types in the deep sea. An important management consideration is whether seamounts should be treated as discrete units, supporting faunal assemblages clearly distinct from other deep-sea habitats. If seamounts are not discrete, then management decisions should be informed by the extent to which seamounts overlap with other habitats. In sections 2.1 and 2.3 it was discussed that seamounts are now believed to have lower rates of endemism than previously thought, and seamount species in most situations are drawn from wider regional pools [4]. However, these findings are still challenged by the relatively small numbers of seamounts sampled, the limited amount and type of sampling carried out on individual seamounts, the over-representation of certain regions of the oceans in seamount studies, and the small, though increasing, number of studies that investigate biodiversity across habitats e.g. [17]. Also, even when the species composition of seamounts may resemble that of other habitats, they can support higher biomass [20] (potentially supporting the bulk of the population of a species) and species of high vulnerability [72] making them prime conservation targets.

Future seamount research programmes must broaden their focus to wider deep-sea communities in order to understand their regional significance, and include habitats such as the continental slope, canyons, and sites of hydrothermal venting or methane seeps that host chemosynthetic communities. Successful deep-sea management regimes will need to consider a suite of biological systems in a regional framework.

In expanding sampling effort to habitats surrounding seamounts, there is a challenge to improve standardisation of sampling gear and survey design which is required for valid comparisons. This may not always be possible but should be consistent within regions where the same sampling gear at least can be widely used.

#### 3.5 Understand the broader effects of human disturbance

The direct physical impact of human activities on seabed communities can be observed and measured (though additional well-designed studies are needed). However, management needs to consider a wider range of impacts than just those in the immediate area of disturbance, because of the likelihood of ‘downstream effects’. If such spill-over impacts are spatially prominent and found to be detrimental to the biota abutting the actual physical impact zone, spatial conservation planning will have to incorporate buffer zones around fishing or mining areas. This will be especially relevant in current fisheries management strategies that rely heavily on closing areas to protect marine ecosystems [113], [114].

Bottom trawling and mining cause direct physical changes to the seafloor and biota; these impacts are now well documented [71], [72], [75], [115]. Conversely, indirect effects and impacts that occur outside the area of direct physical disturbance are poorly understood. Such impacts are predicted to be mainly caused by sediment plumes generated during the trawling and mining operations. It is believed that sediment plumes can remain suspended at abyssal depths for long periods and dissipate very slowly [111]. Higher current flows around some seamounts may disperse plumes faster, but, although modelling work on dispersal rates has been carried out [116], there has been very little experimental work done on this aspect. The spatial scale of such effects is unknown and needs to be determined, both on seamounts and in other deep-sea habitats.

Summaries of information on seamount trophic architecture indicate that they comprise a diverse array of pelagic and benthic consumers [1], [78] often with elevated biomass compared with the surrounding ocean [20]. Changes in the relative abundance of species on a seamount can almost certainly influence trophic linkages and the overall structure of the system, yet few detailed trophic studies have been conducted on seamount communities. Of particular concern are large-scale removals of filter-feeders such as corals and sponges that can dominate the benthic invertebrate assemblages [117]. However, also important is the potential depletion of predator populations where the consequences of overexploitation of low productivity taxa (such as sharks) on ecosystem structure and function are increasingly recognised in shallow waters [118]. These types of indirect effects from trawling or longline operations are uncertain, and should be addressed.

#### 3.6 Quantified recovery dynamics

A common management option to mitigate the impacts of human activity is to close a seamount to fishing or mining [76]. However, whether such action is effective depends upon whether seamount communities will recover to their original state.

The immediate physical and ecological impacts of human activities (e.g. bottom trawling, mining) on the benthos of seamounts are now well documented [49], [71], [72]. By contrast, although some seamounts have been closed to fishing for several years, medium- and long-term effects of fishing are uncertain [86], [119]. Benthic assemblages on seamounts that were closed to fishing for 5 years in New Zealand and for 10 years in Australia had not recovered to any measurable extent, suggesting that seamount biota are likely to take very long periods to revert to pre-fishing conditions [81]. It remains, however, uncertain what the rates of recovery are, how community composition changes over time following cessation of disturbance, which species recruit early to disturbed patches, how fast newly recruited species grow, and whether structurally complex habitats formed by deep-water corals can return. Thus, it is important to increase studies on connectivity between seamounts, recruitment and recolonisation rates, and the age and growth of benthic fauna. The most practical way forward, however, is to undertake time-series surveys to measure changes in the seamount communities over many years. New research needs to be developed, as well as to continue existing time series, such as the ones on the Tasmanian seamounts off Australia [72] and the ‘Graveyard” seamounts off New Zealand [74].

#### 3.7 Determine the effects of climate change

There are numerous potential impacts of climate change on the marine environment as conditions alter; such as temperature, CO_2_ levels, ocean circulation, O_2_ levels, and primary productivity. These can affect all deep-sea communities, although seamount habitat is perhaps particularly relevant to the first two conditions.

The presence of so-called living fossils has been taken as support for the contention that seamounts formed refugia from past dramatic environmental change [13], [78], [120]. For terrestrial species, survival in a globally warming environment is thought to partly depend upon the existence of a nearby “cool refuge” [121], and hence it is possible that the deeper and cooler waters of seamount slopes could act as refugia for benthic fauna from the effects of ocean warming.

Other likely future impacts upon the marine environment resulting from elevated CO_2_ emissions include the consequences of ocean acidification for benthic communities [122]. For example, Guinotte, et al. [123] predicted that, by the end of the 21^st^ century, shallowing of the aragonite saturation horizon (ASH) could leave the majority of deep-sea stony corals in water unsuitable for building their carbonate skeletons. However, Tittensor, et al. [124] found that the effects of ocean acidification on suitable coral habitat, whilst dramatic, are likely to be less pronounced for seamounts than for other deep-sea habitats. Their model predicted that some seamount summits will occur in water better saturated with aragonite and remain as suitable habitat for coral, and thus may act as ‘shallow-water’ refugia for stony corals from the detrimental effects of ocean acidification at greater depth (particularly in the North Atlantic).

The seamount refuge hypothesis depends upon the ability of organisms such as corals to disperse vertically. A recent study by Miller, et al. [24] on the genetic population structure of the deep-sea coral *Desmophyllum dianthus* showed that corals from different depth strata (even on the same or nearby seamounts) were strongly differentiated, indicating limited vertical larval dispersal. Although the reasons for this depth stratification are unclear, it could mean that deep populations of such corals are unable to colonise shallower water at the seamount summit as the ASH rises and deep waters become uninhabitable. Similarly, deep waters might not act as refuges for shallow populations that are impacted by higher temperatures.

Thus, while seamounts may have acted as faunal refuges over historical time-scales, it appears equivocal as to whether they will provide a similar function over the time scale that current climate change and ocean acidification effects are likely to operate (<100 years). Climate change effects are multiple in the ocean (warming, current flow pattern changes, stratification etc) and can operate synergistically with other human impacts (e.g. fishing) [125]. Clearly more research is required to determine the impact of increased levels of atmospheric CO_2_ upon ocean fauna, including those found at seamounts.

### 4. Future data and tools for seamount conservation and management

In the sections above we have described seven research areas that we believe need to be progressed to inform managers and management agencies. These research focus areas will provide important scientific information necessary to improve our understanding of seamount structure and function, and hence gain better insight into the efficacy of various management and conservation options.

However, there are additional data sets and analysis techniques that we feel should be developed and applied to support a range of research and management objectives, and we describe these in the sections below (and see Table 2).

**Table 2:** Summary of resource priorities for seamounts over the next decade based largely on science input required for the growing demands of conservation and management strategies to be developed for seamount ecosystems; no ranking of priorities is implied.

Rationale	Actions	Output(s)
**Seamount data and information**		
Recent international efforts have compiled physical and biological data on seamounts at regional and global scales. These enabled analyses that have improved our understanding of the drivers of faunal assemblages on seamounts, and their spatial distribution. However, many more data are available for inclusion in these databases.	Expand and maintain regional and global databases that document seamount fauna and physical characteristics (e.g. SeamountsOnline, Seamount Catalog).	Data in these databases can be used in a variety of analyses (e.g. biogeographic patterns , environmental classifications) that can contribute to spatial planning strategies for the conservation and management of seamounts.
**Fisheries data and information**		
Development of effective fisheries management requires catch and effort data that cover all major operations and geographic areas and identify individual seamounts catches.	Capture historical data sets into existing global repositories, and improve the spatial resolution at which data are reported.	The detailed distribution of fisheries, and hence impacts on seamounts can guide conservation efforts. Fisheries stock assessment is improved with better data.
**Predictive species distribution modelling**		
Biodiversity maps will for the foreseeable future remain incomplete due to limited sampling coverage. Predictive modelling can extrapolate biodiversity across large ocean scales.	Produce models of species and assemblage distribution as data compilations become available.	Better maps on biogeography are used for management purposes. Models likely to be especially useful for taxa of particular management interest.
**Environmental surrogates**		
Biological sampling of seamounts will remain sparse, so alternative approaches that provide surrogates for biodiversity are needed.	Determine the extent by which physical and chemical parameters can predict biological information, and test the validity of surrogacy models.	In the absence of biodiversity information, managers should be able to use classifications and other measures of surrogacy,
**Risk assessments**		
There is an increasing need for the provision of ecological risk assessments (ERA) for seamounts, as environmental managers attempt to understand the threats posed by fishing and mining.	Refine ERA methods so that they are robust, transparent, and understandable. Assessments tailored to management objectives and available data.	ERAs should facilitate the effective management of seamount resources and inform conservation strategies.

#### 4.1 Seamount data and information

Recent years have seen improved collation of global data on seamounts and other deep-sea habitats through initiatives such as CoML and national science projects directed at the establishment of research networks e.g. [6], [11]. The momentum these projects have created amongst the seamount and deep-sea scientific communities should be maintained, and in particular the sharing of data that has improved dramatically. Many of the advances in scientific understanding enabled by these programmes has come about through the analysis of global or regional-scale data [5].

There are two major examples of global-scale seamount-focused databases:

1. The biogeography database SeamountsOnline, which contains species records specifically from seamounts compiled from a wide variety of databases, survey records, published reports and papers [126] (http://seamounts.sdsc.edu). This database has full taxonomic information, and includes detailed sampling effort information as well as species records, and hence can enable a wide range of analyses. SeamountsOnline has also provided data to the Ocean Biogeographic Information System (OBIS. http://iobis.org), although the latter hosts only a subset of the information (species, location, depth). The database currently has information from about 300 seamounts, with over 20,000 individual faunal records.
2. The geological compilation SeamountCatalog [127] (http://earthref.org). This database holds physical information on seamounts, including location, height, volume, and shape, as well as processed bathymetry and contour maps of each seamount. It currently contains maps and corresponding data from over 1800 seamounts.

Such datasets can inform global-scale analyses and act as a long-term resource for science research and planning, yet lack long-term funding. In addition to maintenance costs, there are still numerous national or regional datasets that can be entered into SeamountsOnline, and increasing amounts of multibeam-derived bathymetry of seamounts into SeamountCatalog, and hence the long-term operation of such global databases need to be supported.

#### 4.2 Seamount fisheries data and information

In order to conserve and manage seamounts, particularly on a global scale and for the high seas, it is necessary to understand which seamounts in which regions have already been fished, and the extent of that fishing effort. Site-specific data, ideally from individual tows is recognised as a requirement for undertaking robust stock assessment of deep-sea fisheries [89]. In addition, without this knowledge it is not possible to assess the impact that may have already occurred, nor the vulnerability or risk of seamounts to fisheries-driven disturbance [55].

Several compilations of fisheries data for seamounts have been undertaken but these have not been at an appropriate spatial scale to confidently associate catch and effort with individual seamounts [50], [66], [128]. Several Regional Fisheries Management Organisations (RFMOs) are attempting to collect data at such a scale for regional management under the FAO guidelines for deep-sea fishing in the High Seas [89]. However, global knowledge of trawl distribution on seamounts remains poor over large areas of the ocean. National fisheries logbook records and vessel monitoring system data [129]–[131] can be used to complement regional and global data compilations.

Two initiatives can improve the data situation:

Firstly, wherever possible, historical data, in particular those from nations with major distant-water fishing fleets (e.g. USSR, Cuba, China, Korea, Japan) need to be compiled and entered into existing databases. The FAO catch system is the primary data repository globally, but it does not contain records from the largest seamount fishery - the Soviet and Japanese fishery in the 1980 s for armourhead and alfonsino on the Emperor and Hawaiian seamount chains. This task should be done urgently, because catch records from the 1960–1980 s are unlikely to be in electronic format, and could be lost forever as institutional ‘memory’ fades over time.

Secondly, catch and effort data should be reported at the scale of individual seamounts, and, ideally, at the finer scale individual tows/sets, as this would make it possible to assign catch and effort to individual seamounts. This level of location detail is routinely recorded on fishing vessels, but is rarely reported to fishery agencies at that level of precision. Analysis of catch per unit effort data is a common method for deep-sea fish stocks, and is regularly used where no fisheries-independent information exits [132]. Such data would enable management agencies to monitor changes in the degree of fishing success, which arguably can be interpreted as indicative of changes in relative abundance of the target fish and bycatch species [68]. It will also clarify movement of the fishing operations on a scale necessary to monitor environmental impact, by highlighting areas where fishing is occurring repeatedly compared with areas where effort (and hence fishing impact) is minor, or if fishers are moving from one seamount to another in turn, serially depleting the fish populations [133].

#### 4.3 Predictive species distribution modelling

Seamount management, especially in areas well offshore, is restricted by the quality and quantity of scientific information. The large number of seamounts in the world's oceans makes it physically impossible to sample even a moderate proportion of them. Even if detailed surveys were possible, these would only cover a few seamounts, and likely take several years to complete. Hence science input to management and conservation initiatives often has to be based on limited data.

Recent advances in three areas have improved our capabilities to predict species occurrences in regions for which no species records exist: 1) new comprehensive global datasets of species occurrences (e.g., cold-water corals) and their habitat associations; 2) finer resolution of environmental data layers that can be correlated with biological data, and 3.) the availability of analytical methods better suited for presence-only data (e.g., Environmental Niche Factor Analysis, Maximum Entropy modelling, Boosted Regression Trees).

Predictive modelling is a useful tool for generating species distribution based on environmental conditions under which species are likely to occur. The technique has been successfully applied to cold-water corals on an ocean-basin or global scale [134], [135] as well as smaller regional studies [136]–[138]. This method appears to work well over broad areas such as the continental slope, but there are issues with the small size of seamounts and the scale of environmental data [42]. It is also critical to determine which environmental data are most important in determining the abundance, as well as the distribution, of a species or taxon, and to more rigorously ground-truth model outputs.

Biodiversity maps will, for the foreseeable future, remain incomplete due to limited sampling coverage compared with the vastness of the oceans. Thus, predictive modelling is valuable to extrapolate species richness (e.g. demersal fish [139]) and assemblage composition (e.g. ophiuroids [140]) beyond the physically known and should be applied to other taxa as data compilations become available.

#### 4.4 Environmental surrogates

A possible solution to overcome a dearth of biological data is to use physical, geological and chemical surrogates for biological information. Environmental parameters (e.g. water chemistry, bathymetry, seafloor composition) are often better known at the scale of ocean-basins than biological information. Thus, environmental ‘proxies’ that have some biological meaning can be used to generate first-cut, approximate estimates of biodiversity to inform spatial management [44]. Examples are two studies that have used biologically-meaningful environmental variables to group seamounts into a seamount-scale classification (Rowden, et al. [141] for New Zealand, and Clark, et al. [142] for the global ocean), giving managers an idea of the scale of potential management for benthic communities, and the type of management option that may be appropriate to achieve certain objectives.

However, the relationship between deep-sea communities and habitat descriptors is complex and ill-resolved for many regions and assemblages. Thus, while environmental surrogates can be useful, they remain proxies, often of unknown accuracy, for actual biological data [8], [143]. A greater emphasis on “ground-truthing” regional-scale studies is necessary to improve confidence in applying the results of these techniques.

#### 4.5 Risk assessments

Risk assessment approaches and analyses can also be applied to identify seamounts that could be most at risk from human impacts.

Clark & Tittensor [55] combined data on seamount physical characteristics, fishery depth and geographical location with a habitat suitability index for stony corals to derive an estimate of the relative vulnerability of the seamount benthos to bottom trawling. Other approaches can be applied using expert knowledge to inform “first-cut” risk assessment [144], or developed further depending on the amount of data available (e.g., Ecological Risk Assessment for the Effects of Fishing (ERAEF) method of Hobday, et al. [145]). A risk assessment framework with a hierarchical structure enables higher-risk interactions to be identified and prioritised in the early and intermediate assessment stages by screening out lower-risk interactions. The ERAEF method was applied using data from biodiversity surveys and knowledge of commercial fishing patterns to evaluate risk to benthic habitats from bottom trawling for a group of seamounts off New Zealand [146]. Although this sort of method can become very detailed, where data are lacking inferences can be drawn from other areas or ecological theory, and can provide a way to assess risks to marine habitats in a rigorous, transparent, and repeatable manner [147]. Risk assessment frameworks can also serve to highlight where critical gaps in knowledge occur that could help prioritise future research.

## Discussion

There are many aspects of seamount and deep-sea ecosystem structure and function that we do not understand, and which may in the long-term be critical for effective management. However, research is in many respects still at the stage of describing the composition and structure of seamount habitat and communities, and appreciating complex functional processes is still a long way in the future. Arguably, we have proposed that the priorities for science that can best inform management are at present to describe structural patterns over various spatial scales, rather than in-depth studies of a small number of seamounts (refer summary of these elements in Table 1). Ideally, future research can comprise a combination of broad scale as well as detailed studies, but the former is needed to plan for the latter if it is to be achieved.

Better science is one thing, but just as critical is the transfer of this information into robust advice which is in a form that managers can readily understand and use. A key element for this to happen is close cooperation and collaboration between scientists, managers, policy agencies, commercial companies, and NGOs at the outset when planning research [148]. The international network of scientists created by the CenSeam programme facilitated communication because of the extensive linkages between managers and scientists in various countries, and ready access to the large amount of data and information within the network [5]. This should happen as a matter of course because much seamount research today is funded by management agencies that are responsible for regulating mining or fishing activities. However, it appears that too often there is a lack of understanding between scientists and managers about what is required, or the appreciation that basic descriptive research is needed to underpin more applied objectives.
